# Cancer therapy by restoration of immune Notch

**DOI:** 10.1186/2051-1426-1-S1-P88

**Published:** 2013-11-07

**Authors:** Anil Shanker, Duafalia F Dudimah, Roman V Uzhachenko, Asel K Biktasova, David P Carbone, Mikhail M Dikov

**Affiliations:** 1 Department of Biochemistry and Cancer Biology, Meharry Medical College School of Medicine, Nashville, TN, USA; 2 Department of Cancer Biology, Vanderbilt-Ingram Cancer Center, Vanderbilt University, Nashville, TN, USA; 3 Department of Medicine, James Cancer Center, The Ohio State University, Columbus, OH, USA

## 

Factors affecting host immunity are important variables during cancer growth. The immunosuppressive tumor microenvironment perturbs numerous immune regulatory networks and usurps host antitumor immune responses. We discovered that tumor interferes with host hematopoietic Notch system. Tumor-induced decrease in Notch signaling in host immune cells could be a major causative link in the inadequate induction of anti-tumor immunity. Interestingly, we found that tumor-induced decrease in immune Notch could be restored therapeutically by the following two agents. Administration of a novel Delta-like ligand 1 (DLL1) multivalent cluster, and FDA-approved proteasome inhibitor drug bortezomib - which also sensitizes tumors to death signals - could activate Notch 1 signaling in lymphoid cells of tumor-bearing mice without increasing tumor cell proliferation or clonogenicity. Systemic activation of DLL1-Notch signaling could attenuate tumor vascularization as well as increase T cell infiltration in tumor, decrease proportion of regulatory T cells and enhance antitumor T cell function and memory in multiple mouse tumor models. This resulted in significant suppression of tumor growth in wild type mice as well as provided therapeutic benefit following an adoptive T cell transfer into tumor-bearing SCID-NOD mice. New data also show that bortezomib affects the expression of notch receptors and ligands differentially in lymphocytes and in a wide range of solid tumor cells: 4T1, PyMT, MDA-MB-231, C26, LLC, D459, and Renca-HA. Moreover, bortezomib administration increased the expression of Notch target Hes1 in thymus, lymph node and spleen of tumor-bearing mice, suggesting a potential synergistic action of bortezomib and DLL1 activation of Notch signaling. This new role of Notch in the development of effective antitumor immunity and the potential of its modulation by a novel prototypic agent DLL1 in combination with bortezomib present exciting opportunities to uncover multi-pronged immune stimulatory regimens. Reagents based on the multivalent forms of Notch ligands thus need to be explored for the therapeutic modulation of Notch signaling. Therapeutic restoration of immune Notch signaling could provide effective treatment and recurrence-free survival in cancer patients by breaking tumor resistance and induction of robust antitumor immunity.

